# Clostridium perfringens Empyema: Anaerobic Invasion in an Uncommon Location

**DOI:** 10.7759/cureus.60082

**Published:** 2024-05-11

**Authors:** Harjinder Singh, Jessica Kaushal, Alejandro Garcia, Vivek Kak

**Affiliations:** 1 Internal Medicine, Henry Ford Health System, Jackson, USA; 2 Internal Medicine, Bridgeport Hospital, Bridgeport, USA; 3 Infectious Disease, Henry Ford Health System, Jackson, USA

**Keywords:** pulmonary decortication, decortication, antibiotics, sepsis, pulmonary embolism, empyema

## Abstract

*Clostridium perfringens* bacteremia arises due to skin inoculation from the external environment or translocation from the gastrointestinal tract. In the event of bacteremia, it tends to colonize in anaerobic environments due to its obligatory anaerobic nature. Its inoculation in the lung, albeit rare, can occur if an anaerobic nidus is created. In the presented case, the patient developed *C. perfringens *bacteremia andempyema in the area of lung necrosis caused by acute pulmonary embolism. He did not have any history of chest trauma, and the source of bacteremia was deemed to be via gut translocation. The patient was noted to have multiple gastric ulcers on endoscopy and jejunal wall thickening, which likely led to the bacterial translocation into the bloodstream. He underwent video-assisted thoracoscopic surgery-assisted decortication and intravenous antibiotics, eventually leading to clinical improvement. To identify the source of *Clostridium* in the absence of penetrating trauma, a thorough gastrointestinal evaluation, including a colonoscopy, is warranted to identify the pathology leading to the gastrointestinal translocation.

## Introduction

*Clostridium perfringens*, a widely distributed bacterium belonging to the *Clostridium* family, was first described in the late 1890s in association with the cases of gas gangrene [1]. While *C. perfringens* has been commonly associated with gas gangrene and enterocolitis in neutropenic patients throughout modern history, its involvement in pleuropulmonary disease is rare.

The bacterium is usually believed to cause disease through trauma-related inoculation by environmental spores or foodborne toxin transmission. Dissemination via bacteremia and subsequent invasion to other sites are rarely reported, and when a source is identified, the gastrointestinal system is typically implicated as its origin [2]. Moreover, the occurrence of pleuropulmonary disease secondary to bacteremia is exceptionally rare, with few reported cases attributed to gastrointestinal pathologies such as bowel obstruction, metastatic focus seeding, liver disease, or malignancy [3-5]. *C. perfringens-*related pleuropulmonary disease almost certainly presents with necrotizing pneumonia complicated by parapneumonic effusions and empyema.

Here, we present a patient with *C. perfringens* bacteremia, which likely translocated from the gastrointestinal source, colonized the necrotic lung tissue caused by pulmonary embolism, and led to necrotizing pneumonia with empyema. 

## Case presentation

A 63-year-old male with a past medical history of paroxysmal atrial fibrillation, left ventricular thrombus, and non-ischemic cardiomyopathy with an ejection fraction of 24% presented to the emergency room complaining of cough with purulent sputum production. His cough started insidiously around two weeks ago with associated thick yellow sputum. He also had generalized weakness for the same duration of time to the extent of making him bedbound. He was prescribed rivaroxaban for the left ventricular thrombus but was not compliant with it for over a week due to his illness.

**Figure 1:**
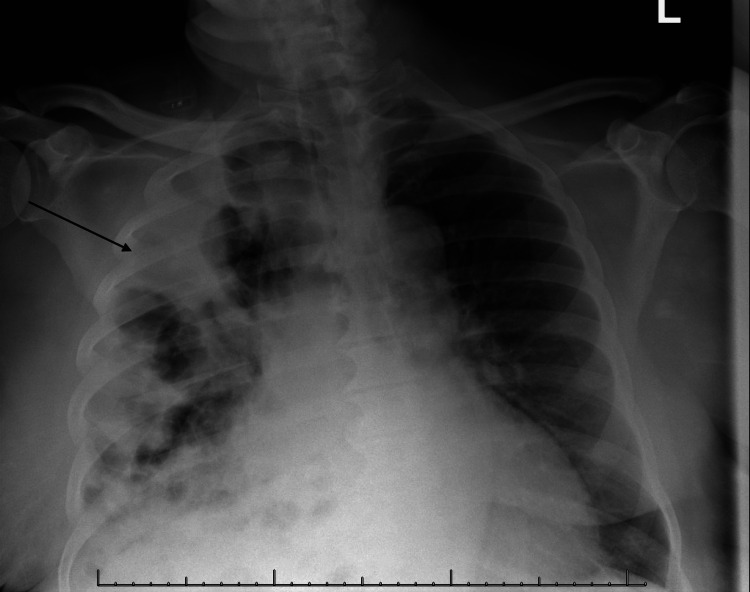
Chest X-ray showing multilobar opacities in the right lung fields (arrow)

**Figure 2:**
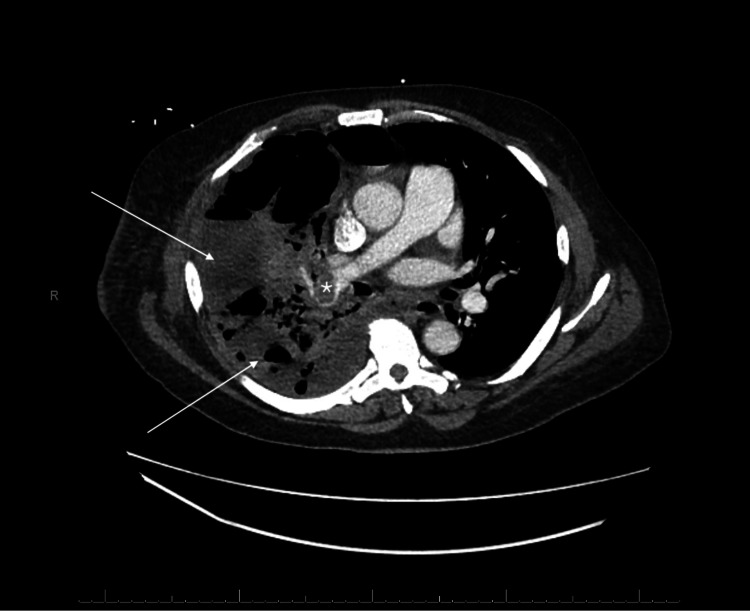
Chest CT with intravenous contrast showing acute pulmonary embolism in the right pulmonary artery (star) with extensive areas of necrotizing pneumonia and associated empyema (arrows)

**Figure 3:**
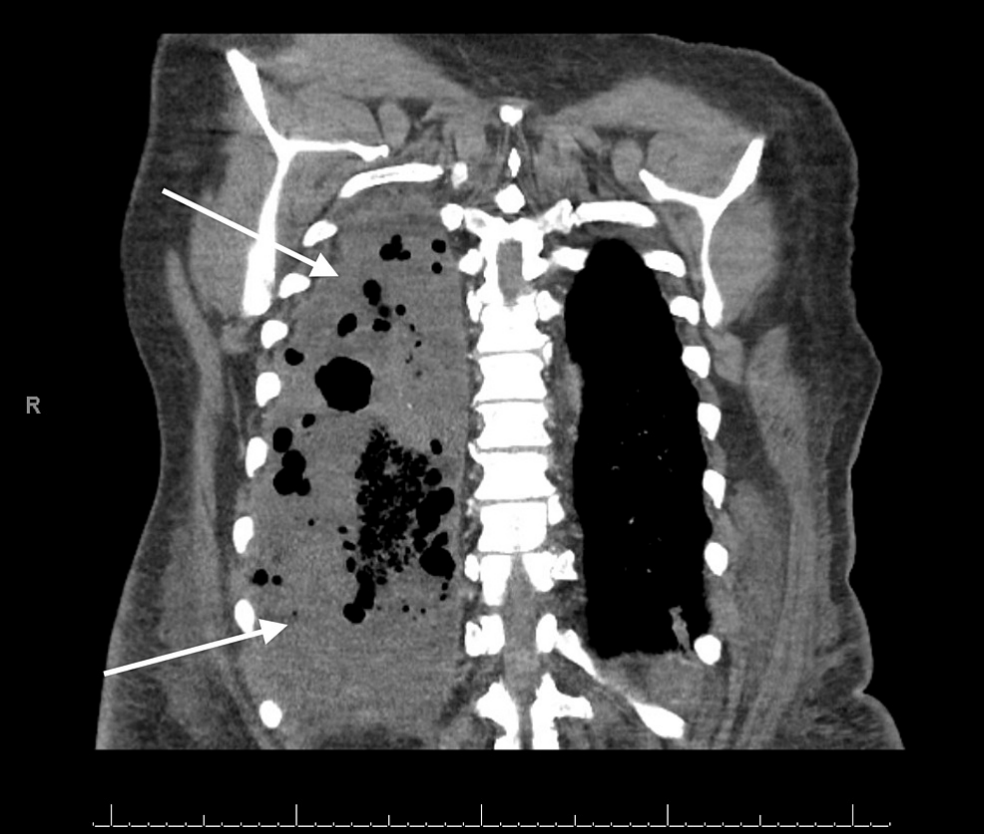
Coronal view of the chest CT showing multilobar opacities on the right side (arrows) depicting the extent of necrotizing pneumonia

On examination, the patient was ill-appearing and hypoxic with an oxygen saturation of 88% on ambient air, which improved to 97% on 2 liters/minute oxygen via nasal cannula. He had a temperature of 36.1°C, blood pressure of 127/113 mm Hg, heart rate of 102 per minute, and respiratory rate of 24 breaths per minute. On auscultation, he had decreased breath sounds on the right-sided lung fields with normal breath sounds on the contralateral side. Laboratory workup showed leukocytosis of 17,400/μL with 85% neutrophils and 2% immature granulocytes. Chest X-ray showed multilobular opacities in the right-sided lung fields (Figure 1), suggesting consolidation or empyema. Chest CT with intravenous contrast showed acute pulmonary emboli (PE) within the right upper, middle, and lower lobar, segmental, and subsegmental pulmonary arteries and smaller subsegmental emboli in the left upper and lower lobe pulmonary arterial branches. It also showed extensive multiloculated right pleural effusion with numerous internal locules of gas, multiple cystic spaces, and emphysematous bleb formation concerning necrotic infection (Figure 2, Figure 3). Transthoracic echocardiogram showed an ejection fraction of 30%, a severely enlarged right ventricle (RV), a severe tricuspid regurgitation, a moderately reduced RV systolic function, and no evidence of RV strain. The lower extremity Doppler ultrasound revealed an acute occlusive deep venous thrombosis (DVT) in the bilateral peroneal veins and right distal thigh varicose vein. His prolonged immobilization due to generalized weakness was thought to be the contributing factor to the thromboembolism.

**Figure 4:**
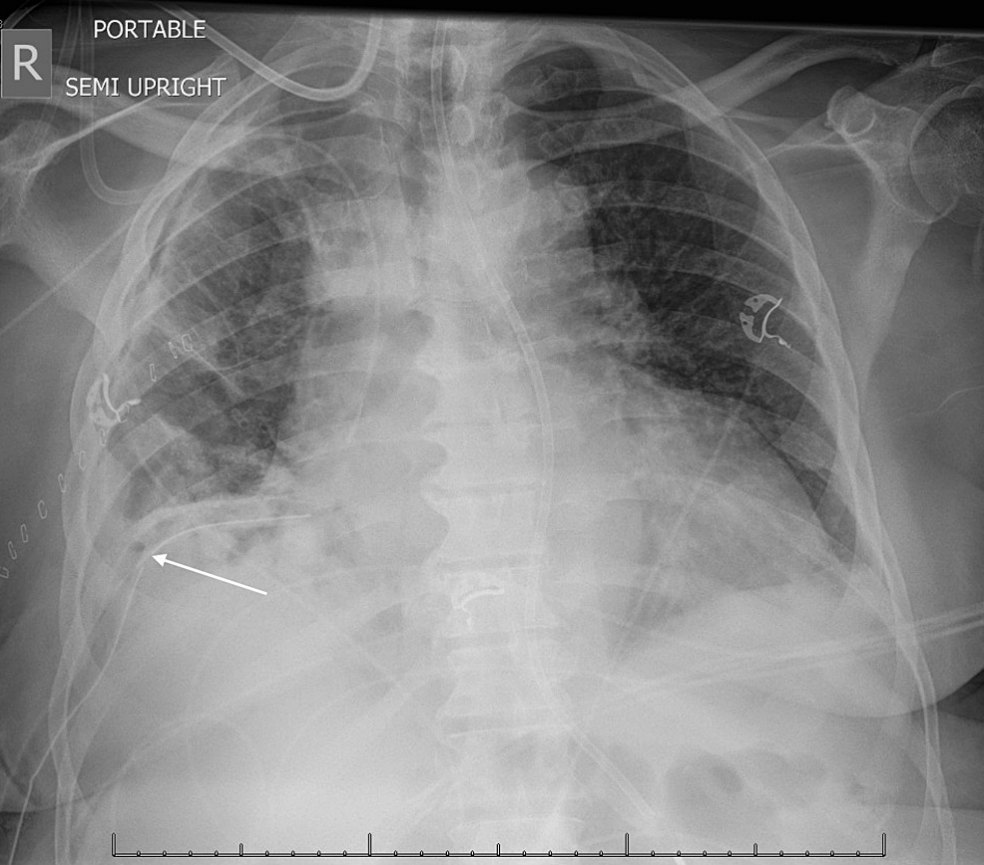
Post-surgery chest X-ray showing improvement in multifocal lung opacities with the chest tube (arrow) in place

The patient was started on unfractionated heparin (UFH) for pulmonary embolism and DVT and intravenous vancomycin, cefepime, and metronidazole for the empyema. He underwent right open thoracotomy with decortication, surgical evacuation of lung abscesses, and placement of chest tubes. Postoperatively, the patient was transferred to medical intensive care as he required vasopressor support and mechanical ventilation for respiratory failure. After two days, his mentation and shock improved, and he was extubated successfully. His blood and intraoperative cultures grew penicillin-susceptible *C. perfringens, *and the antibiotic regimen was de-escalated to ampicillin-sulbactam. Post-surgery chest X-ray showed interval improvement in multifocal opacities seen previously on the admission chest X-ray (Figure 4). 

The patient's hospital stay was complicated by melena and anemia, requiring 13 units of packed red blood cell transfusion. Esophagogastroduodenoscopy showed a large 20-millimeter-sized non-bleeding gastric ulcer in the gastric fundus just distal to the gastroesophageal junction, presumed to be the culprit of the anemia and bleeding. There were also multiple smaller-sized, non-bleeding gastric ulcers as seen on endoscopy. Workup of hemolytic anemia was pursued due to the possibility of *Clostridium*-induced hemolysis. He had an elevated haptoglobin of 290.1 mg/dL (normal 30-200 mg/dL) and an elevated lactate dehydrogenase (LDH) level of 319 IU/L (normal 100-220 IU/L). Peripheral blood smear showed normochromic normocytic anemia without any suggestive signs of hemolytic anemia. The patient did not have any penetrating chest wall trauma or thoracic wall procedure to account for the bacterial contamination of the pleura. CT of the abdomen and pelvis showed severe wall thickening in the visualized jejunum, which was presumed to have led to gastrointestinal translocation, given the prolonged illness.

Due to the high risk of bleeding and the requirement of anticoagulation for PE, an inferior vena cava filter was placed, and anticoagulation was continued. The patient returned to his baseline functional activity and was discharged to subacute rehab. His chest tube was continued, ampicillin-sulbactam was transitioned to ampicillin-clavulanate, and UFH was changed to apixaban with plans for close outpatient monitoring. 

## Discussion

*Clostridia* are rarely responsible for lung infections in non-surgical or non-traumatic scenarios. They are not typically the main culprits in anaerobic empyema and are commonly found in the presence of other aerobic or anaerobic bacteria [3]. *C. perfringens* is typically present on the skin or gastrointestinal tract as a normal component of the gut microbiota, albeit in low quantities ranging up to 103 colony-forming units per gram [6]. There was a well-established history of heart failure with a reduced ejection fraction of 24% and right ventricular dysfunction, which could have contributed to systemic congestion and inflammation, leading to disrupted gut microbiota, heightened intestinal permeability, and functional dysbiosis of the gut [7]. Certain factors that increase the likelihood of developing pleural empyema from *Clostridium* bacteremia include pulmonary infarction from aspiration pneumonia, necrotizing malignancies, and pulmonary embolization [8]. Our case did not have a history of any trauma, surgery, or iatrogenic contamination of the pleural space to explain the translocation from the skin surface. In the context of our patient, extensive lung necrosis from pulmonary embolism created a favorable environment for the anaerobic growth of *C. perfringens*. The patient had no history of gastrointestinal malignancy, commonly linked to this bacteremia [6]. Its presence was not entirely excluded as the patient had never undergone a colonoscopy or any stool tests for colon cancer screening.

*C. perfringens* has been associated with producing 20 toxins, its main virulence factors. Based on this characteristic, strains of *C. perfringens* can be categorized into seven toxinotypes (A-G) depending on their type. The probable toxins leading to this empyema may have included alpha toxin and perfringolysin O, which are crucial in histotoxic infections, along with epsilon toxin, which is associated with increased vascular permeability to facilitate blood-tissue bacterial translocation [8]. These toxins likely allowed for invasive infection to an already necrotic lung parenchyma tissue. Apart from gastrointestinal translocation, oropharyngeal aspiration may have also played a role in this context. However, *C. perfringens* in the oral mucosa is uncommon, as one study showed its colonization in only 12% of hospitalized patients [9]. This environmental predisposition, coupled with the diverse toxins produced by *C. perfringens*, results in necrotizing pneumonia and is invariably accompanied by effusion or empyema. The alpha toxin, a phospholipase C, is known to cause the aggregation of platelets, leukocytes, and fibrin in the blood vessel and lead to clot formation [10], as one study showed a rapid and irreversible decline in skeletal muscle perfusion on intramuscular injection of phospholipase C molecule [11]. This gives an idea about the possibility of* Clostridium *sepsis causing the pulmonary embolism and subsequently inoculating in the anaerobic environment. A workup for intravascular hemolysis is warranted in developing anemia in *C. perfringens* bacteremia. It is seen in 7-15% of cases of *C. perfringens* bacteremia and is associated with a significant mortality rate of 80% [12]. In this case, the cause of anemia was likely from gastrointestinal bleeding instead of hemolytic anemia due to no evidence of hemolysis on the peripheral blood smear. The elevation of LDH is presumed to be a result of an acute-phase reactant response.

To our knowledge, this is the third case of pulmonary embolism complicated by *C. perfringens* empyema. The three previous cases ended in fatal outcomes after being complicated by septic shock [13,14]. Despite the poor prognosis of *C. perfringens* empyema, with some series reporting 30% mortality [4], cases associated with pulmonary embolism present an unusually high mortality within this group. In previously reported cases, there were variations in antibiotic management and surgical approaches. Antibiotic regimens included penicillin and metronidazole in one case [13] and isolated penicillin in the other [14]. From a surgical perspective, one case involved immediate chest tube placement [14], while in another case, surgical management was delayed until the patient's condition deteriorated [13]. In our case, the prompt initiation of antibiotics and immediate surgical intervention played a key role in the patient's recovery, as *C. perfringens* is highly susceptible to penicillin [15]. The recommended antibiotic regimen for *C. perfringens* infection is a dual combination of penicillin and clindamycin, as 5% of strains of *C. perfringens* are clindamycin-resistant. Clindamycin also provides the theoretical benefit of the inhibition of toxin production [16]. The efficacy of penicillin has been shown in in vitro studies, and clindamycin has been shown to be effective in animal models [17]. 

## Conclusions

This case underscores *C. perfringens* empyema, an uncommon bacterial isolate from the intrathoracic region. Although often transient and asymptomatic, this anaerobic bacterium can rarely lead to severe complications by inoculating necrotic tissue, as evidenced by the development of necrotizing pneumonia in our patient. A comprehensive gastrointestinal evaluation, including colonoscopy, is imperative in identifying and treating the underlying source of bacteremia, highlighting the need for a multidisciplinary approach in managing such cases.

## References

[1] Grenda T, Jarosz A, Sapała M, Grenda A, Patyra E, Kwiatek K (2023). Clostridium perfringens-opportunistic foodborne pathogen, its diversity and epidemiological significance. Pathogens.

[2] Rechner PM, Agger WA, Mruz K, Cogbill TH (2001). Clinical features of clostridial bacteremia: a review from a rural area. Clin Infect Dis.

[3] Gorbach SL, Thadepalli H (1975). Isolation of Clostridium in human infections: evaluation of 114 cases. J Infect Dis.

[4] Patel SB, Mahler R (1990). Clostridial pleuropulmonary infections: case report and review of the literature. J Infect.

[5] Albuquerque A, Macedo G (2013). Spontaneous bacterial empyema in a cirrhotic patient due to Clostridium perfringens: case report and review of the literature. Gastroenterol Hepatol.

[6] Jackson S, Gregson DB, McFadden S, Laupland KB (2003). Clostridium perfringens pleuropulmonary infection and septic shock: case report and population-based laboratory surveillance study. Scand J Infect Dis.

[7] Uzal FA, Freedman JC, Shrestha A (2014). Towards an understanding of the role of Clostridium perfringens toxins in human and animal disease. Future Microbiol.

[8] Sandek A, Bauditz J, Swidsinski A (2007). Altered intestinal function in patients with chronic heart failure. J Am Coll Cardiol.

[9] Bayer AS, Nelson SC, Galpin JE, Chow AW, Guze LB (1975). Necrotizing pneumonia and empyema due to Clostridium perfringens. Report of a case and review of the literature. Am J Med.

[10] Bashir Y, Benson MK (1990). Necrotising pneumonia and empyema due to Clostridium perfringens complicating pulmonary embolus. Thorax.

[11] Bryant AE (2003). Biology and pathogenesis of thrombosis and procoagulant activity in invasive infections caused by group A streptococci and Clostridium perfringens. Clin Microbiol Rev.

[12] van Bunderen CC, Bomers MK, Wesdorp E, Peerbooms P, Veenstra J (2010). Clostridium perfringens septicaemia with massive intravascular haemolysis: a case report and review of the literature. Neth J Med.

[13] Spagnuolo PJ, Payne VD (1980). Clostridial pleuropulmonary infection. Chest.

[14] Schuetz AN (2014). Antimicrobial resistance and susceptibility testing of anaerobic bacteria. Clin Infect Dis.

[15] Stevens DL, Bisno AL, Chambers HF (2014). Practice guidelines for the diagnosis and management of skin and soft tissue infections: 2014 update by the Infectious Diseases Society of America. Clin Infect Dis.

[16] Bryant AE, Chen RY, Nagata Y (2000). Clostridial gas gangrene. I. Cellular and molecular mechanisms of microvascular dysfunction induced by exotoxins of Clostridium perfringens. J Infect Dis.

[17] Stevens DL, Bryant AE (2017). Necrotizing soft-tissue infections. N Engl J Med.

